# Comparative study of representations of professional autonomy produced by first and last-period undergraduate nursing students ^1^

**DOI:** 10.1590/1518-8345.1919.2927

**Published:** 2017-09-18

**Authors:** Érick Igor dos Santos, Antonio Marcos Tosoli Gomes, Sergio Corrêa Marques, Raquel de Souza Ramos, Aline Cerqueira Santos Santana da Silva, Francimar Tinoco de Oliveira

**Affiliations:** 2 PhD, Adjunct Professor, Departamento de Enfermagem, Universidade Federal Fluminense, Rio das Ostras, RJ, Brazil.; 3 PhD, Full Professor, Faculdade de Enfermagem, Universidade do Estado do Rio de Janeiro, Rio de Janeiro, RJ, Brazil.; 4 PhD, Adjunct Professor, Faculdade de Enfermagem, Universidade do Estado do Rio de Janeiro, Rio de Janeiro, RJ, Brazil.; 5 PhD, RN, Instituto Nacional do Câncer, Rio de Janeiro, RJ, Brazil.; 6 PhD, Adjunct Professor, Departamento de Enfermagem Médico-Cirúrgica, Universidade Federal do Rio de Janeiro, Rio de Janeiro, RJ, Brazil.

**Keywords:** Professional Autonomy, Nursing, Education, Nursing, Social, Psychology, Work, Nurse’s Role

## Abstract

**Objective:**

to compare the social representations of professional nurse autonomy produced by first and last-period undergraduate nursing students.

**Method:**

qualitative, descriptive and exploratory study, based on the structural approach of social representations, the Central Core Theory, carried out with 171 students from three federal public universities, using the free association technique on the object “professional nurse autonomy”. The data were submitted to EVOC 2005 software and to similarity analysis.

**Results:**

care was the central core of the representational structure identified among the students of the first period. Among last-period students, knowledge stood out as a core element. The term responsibility was identified as common to both central cores.

**Conclusion:**

regarding professional autonomy, the results point to an overlapping process of the reified and consensual universes during the undergraduate course. However, responsibility, inherent in the profession, remains cross-sectional. For the first period students, autonomy is resignified in a practical and attitudinal way, whereas for the last period students, the knowledge acquired stimulates them to assign meaning to professional autonomy with a cognitive and attitudinal representation. The data can support the use of innovative teaching practices in nursing undergraduate courses.

## Introduction

In the context of nursing, professional autonomy is a great challenge, characterized by a considerable complexity. Professional autonomy is defined in nursing literature as the autonomy to make decisions in each one’s professional area and to act according to it^1^. It is also related to the ability to make autonomous decisions based on comprehensive understanding about the human being and on knowledge supported by scientific evidence^1^. At the same time, professional autonomy can be defined as independence or self-determination in the professional practice, with legal support to allow the implementation of the decisions regarding nursing actions^2^.

This concept has been associated to the possibility and the capacity for autonomous decision-making, carried out by a personal body of knowledge and ethical obligations with the client. A study conducted with British nurses pointed out the importance of more than one type of knowledge in the daily practice of the participants^3^. Another study, carried out in China, identified that the first step taken by nurses when finding themselves in a decision-making position is to collect information about the patient’s pathology and generating different hypotheses of explanations for the situation. After that, they test each hypothesis independently, using clinical data collected from the patient, in order to identify which hypothesis best applies to the case. Finally, they decide on the most appropriate conclusion for the patient’s health problem^4^.

Autonomy is also seen as a fundamental aspect in the professional practice of the nurse and as a pillar of the quality of care, considering that autonomy in the professional practice provides a higher degree of satisfaction for the nurse, which directly interferes in patient safety. It is defined, therefore, as freedom to act and to make independent clinical decisions in the nursing sphere of practice and interdependent decisions in interdisciplinary spheres, all based on scientific evidences^5^.

A considerable part of the evidence points to the theoretical and conceptual complexity of professional autonomy, as well as to the existence of a philosophical dimension to it. This complexity is possibly due to the fact that researchers have not yet reached an agreement on how autonomy should be defined and understood, and the literature presents incoherent or controversial definitions of the concept, lack of randomized or comparative studies and inappropriate forms to measure it ^(^ ^6^.

Based on studies addressing professional nurse autonomy^1^ ^-^ ^6^, this study aims to fill a psychosocial gap regarding the autonomy of the nurse, unveiling the processes of symbolization and resignification performed by different social groups, such as the students in a nursing undergraduate course. Investigating this group is justified by the scarcity of scientific works addressing the subject of professional nurse autonomy among undergraduate students. Thus, the development of a study connecting the representations and the teaching in undergraduate nursing program is relevant. This study takes in consideration the possibility of constructing a panorama of the professional practice based on the different scenarios through which the undergraduate students circulate, since they are in direct contact with different nurses in several institutions and in different moments over their graduation years. It is based on these meetings and on the intersubjective exchanges with colleagues and professors that students develop a critical perspective on the profession, its know-how and the perception of the (in)existence of their professional autonomy.

Thus, in this study, the objective was to compare the social representations of the professional nurse autonomy produced by nursing undergraduate students from the first and last period, that is, freshman and senior students.

## Method

This is a qualitative, descriptive and exploratory study, based on the theory of Social Representations (SR) according to its structural approach, the Central Core Theory (CCT). For the proposer of the theory, the representation is composed of a determined set of information, beliefs, opinions and attitudes about a social object^7^ ^-^ ^8^. In this perspective, the representation is structured in a specific way, with cognems (words) that form a central core and give meaning to the representation, and peripheral cognems or elements gravitating around the core, which are more accessible and responsible for the concretization, regulation and defense of this core^7^. This approach favors a more objective understanding of the meanings present in the social thought regarding the object of representation. Besides, the use of a projective technique for the data collection favors a more quickly and spontaneous presentation of the semantic meanings given by the students about professional nurse autonomy. 

Data were collected from November to December 2015 in three federal public universities located in the Rio de Janeiro Metropolitan Region, in the State of Rio de Janeiro, Brazil. Two of these universities were located in the city of Rio de Janeiro and one in the city of Niterói. All of them have undergraduate courses in nursing (teaching license) and consolidated graduate programs, nationally and internationally recognized and serving the vast majority of the students approved in a unified selection process for public higher education in nursing.

The study population consisted of 111 students from the first period and 60 students from the last period, totaling 171 participants who were engaged in student activities in the scenarios chosen for the research at the time of data collection. Thus, this research encompasses two distinct subgroups, according to the stage they were in the course. The number of participants was the product of a convenience sample to obtain the required number of participants in the universe of 100% of the students in each class, per academic period. The final sample number, therefore, meets the recommendation studies based on the structural approach of social representations, which is at least 100 individuals^9^.

Nursing undergraduate students attending the following inclusion criteria participated in the study: minimum age of 18 years, without no age limit nor gender distinction. The exclusion criteria were the presence of communication difficulties or inaccessibility of the participant after three consecutive attempts to reach out. No other attribute was a justifiable exclusion criterion.

The regulations from Resolution 466/2012 of the National Health Council (CNS) were followed. These regulations define the ethical and legal aspects of human research, which include the presentation and shared reading of an Informed Consent Form (TCLE), followed by free and conscientious acceptance to participate in the research. As it was an inter-institutional project, the study was approved by the Research Ethics Committees (REC) of the Universidade Federal Fluminense (UFF) and of the Universidade Estadual do Rio de Janeiro (UERJ), obtaining approval under the protocols numbers 924,334 (UFF) and 939,676 (UERJ). 

The free associations technique for the object “professional nurse autonomy” was used, aiming to highlight the semantic universe and the imaginary dimension of the representations. In addition, a questionnaire with closed and open-ended questions was applied in order to characterize the socio-demographic profile of the study participants. The use of the free associations technique preceded the collection of socio-demographic data, so that the latter did not interfere with the data produced. The data collections consisted in asking the students to speak the first five words or expressions that came to mind when they heard the inducing terms. The words were recorded in the order they were recalled. 

As for the data analysis techniques used, a simple descriptive statistical analysis was performed on the data from the questionnaire. Regarding the free associations, a *corpus* with all the words was elaborated, divided by participants and in the order they were recalled, and presented in the four-house board^4^. The *Ensemble des programmes permettant l’analyse des evocations* (EVOC) software, 2005 version, was used to calculate and inform the simple frequency of occurrence of each word, the mean occurrence of each word in the order they were evoked, and the mean of the weighted mean orders of the set of terms evoked (*rang*)^9^ ^-^ ^10^, thus composing the four-house board^9^.

In this table, the upper left quadrant, or central core, contains the words that were evoked more frequent than the number set by the researcher, as well as the most readily evoked, which constitute the possible central elements of the representation. In the upper right quadrant or first periphery are the words which were also very frequent, but not so readily evoked, and which may be reinforcing the central core. In the lower left quadrant, also called the contrast zone, are words that were evoked fewer times than the number set but were promptly evoked. This group may be reinforcing the central core or indicating the presence of a subgroup in the studied group. And in the lower right quadrant or second periphery were the words less evoked and verbalized later, that is, they were mentioned in the last positions^7^ ^,^ ^10^.

Continuing the analysis of the data obtained, similarity analysis of the free associations was conducted in a complementary way. This technique is a procedure used within the structural perspective of social representations, in order to check the number of ties or connections between one element of the representation and another. Its result consists in the construction of a figure called maximum tree or similarity tree. The starting points for the construction of this figure are the highest similarity indices, that is, the strongest connections between words. This technique does not allow confirming the core elements, but it provides another indication of the elements that are central in the representation. Based on the organization of the data on the four-house board and on the similarity tree, the data were analyzed in light of the assumptions of the CCT.

## Results

The participants who were in the first period were mostly females (81%), between 18 and 20 years old (63%), Evangelical (30%), with no personal income (64%), with family income between 1 and 6 minimum wages (62%), with no previous technical training (59%), with no employment bond (94%), who were already attended by nurses when needed (94%) and had access to information about nursing outside the university (86%), mainly via websites (27%). Of those who had prior professional technical training, 55% were nursing technicians. 

Among the students of the last period, the profile was also formed by women (97%), between 21 and 26 years old (90%), Catholic (33%), with personal income lower than one thousand reais (64%), with no previous technical training (62%), with no employment bond (97%), who were already attended to by nurses when needed (67%) and had access to information about nursing outside the university (93%), mainly through scientific journals (52%). Of those who had prior professional technical training, 61% were nursing technicians.

**Figure 1:**
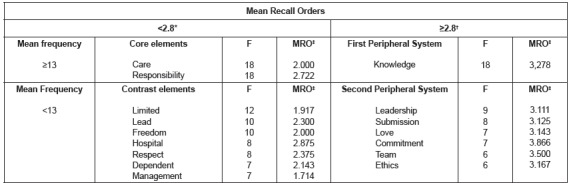
Structure of the social representation of professional nurse autonomy among undergraduate nursing students from the first period (n = 111) of federal public universities in the Rio de Janeiro Metropolitan Region. Rio de Janeiro, RJ, Brazil, 2016.

Regarding the results of the free associations referring to the object “professional nurse autonomy”, among the students of the first period (n = 111), the EVOC software counted 505 words, 238 not repeated. For the purpose of organizing the data, the minimum frequency of 6 words was defined, excluding words with frequency lower than. The mean frequency was 13. The mean of the Mean Recall Orders (MOA), also called rang, was 2.8, calculated by EVOC software on a scale of 1 to 5. Combined analysis of these data resulted in the organization presented in Figure 1.

*<2.8=less than; †≥2.8=greater or equal to; ‡MOA= Mean Recall Order

In the structure of the social representation of professional nurse autonomy, the terms “care” (F=18; R=2.000) and “responsibility” (F=18; R=2.722) were the probable central cores for the group of first-period students. These terms, therefore, determine at the same time the signification and the internal organization of the representation. Thus, they exert a generating and organizing function by creating or transforming the meaning of the other elements of the representation, as well as by determining the nature of the ties that bind the elements of representation together (organizing function)^8^. It should be noted that these terms express a functional dimension (care) and a normative dimension (responsibility) that, in the context of the CCT, are determined by the nature of the object and by the relation that the group maintains with the object. In the functional dimension the practical questions, that is, what is important for the accomplishment of a task, are highlighted; in the normative dimension, questions related to the norm, stereotype, judgment or evaluation, belief, among others are the ones highlighted^7^ ^-^ ^8^.

In the peripheral system of the representation more characteristics of the immediate context of the group appear, that is, it is where the representation is anchored in the reality of the moment of the social group^8^. In this group, the contrast zone contains the terms “limited” (F=12; R=1.917), “lead” (F=10; R=2.300), “freedom” (F =7; R=2.1475), hospital, respect, dependent and “management” (F=7; R=1.714). This result seems to indicate the presence of a subgroup in the group of first period students, since the terms are in contrast with those of the central core. Thus, the words “limited” and “dependent” indicate an evaluative dimension of the group, sometimes restricting the professional autonomy to defined limits, sometimes conditioning it to the institutional context. “Lead” presents itself as a practical dimension that, for the group in question, is the context of the professional practice, in which the professional nurse autonomy is concretized. This idea is reinforced by the term “management”, which links the definition of professional autonomy to the managerial activity. The cognem “hospital” is within the imaginary dimension of the representation, as it may indicate that these students consider that this place of professional activity is where the nurses better show or express their professional autonomy. “Respect” is a concept linked to the group’s value system, indicating the attitudinal dimension of the representation. “Freedom” is an element possibly linked to the cognitive dimension of the representation, since it may indicate a characteristic of professional autonomy. 

The first peripheral system is formed by the term “knowledge” (F=18; R=3.278), indicating another cognitive dimension of the representation. In the second periphery, the elements “commitment” (F=7; R =3.866) and “ethics” (F=6; R=3.116) justify the presence of the term “responsibility” in the probable central core of the representational structure and reinforce the interpretation of the attitudinal dimension as a whole. “Love” (F=7; R=3.143), in turn, appears as the only affective element of the structure, possibly linked to the term “care” of the central core. “Submission” (F=8; R=3.125) reinforces the attitude of the first-period students regarding the object professional autonomy, belonging to their value system on the same object. “Team” (F=6; R=3.500) and “leadership” (F=9; R=3.111) indicate the imaginary dimension of the representation, where autonomy is expressed.

**Figure 2:**
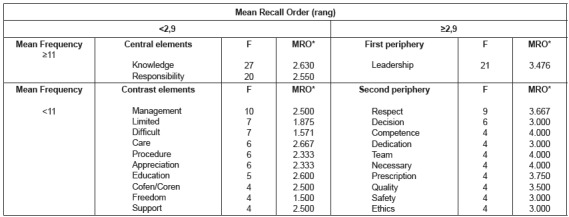
Structure of the social representation of professional nurse autonomy among undergraduate nursing students from the last period (n = 60) of federal public universities in the Rio de Janeiro Metropolitan Region. Rio de Janeiro, RJ, Brazil, 2016

According to Figure 2, 301 words were mentioned by the last period students period (n = 60), regarding the object “professional nurse autonomy”, 86 of them not repeated. For the purpose of data organization, the minimum frequency of 4 words was defined and the mean frequency was 11. The mean of the Mean Recall Orders, also called rang, was 2.9, as calculated by the EVOC software on a scale of 1 to 5. The combination of these data generated the picture shown in Figure 2

*MRO= Mean Recall Order.

The probable central core of the social representation of professional nurse autonomy, for last period students, is formed by the terms “knowledge” (F=27; R=2.630) and “responsibility” (F=20; R=2.550). The first is related to the cognitive dimension, of functional nature, and the other to the attitudinal dimension, of normative nature. The terms “limited” (F=7; R=1.857) and “difficult” (F=7; R=1.571) in the contrast zone and “necessary” (F=4; R=4.000) in the second periphery reinforce the meaning of the term “responsibility” in the probable central core and are within the evaluative dimension of the representation.

It is possible to identify elements that are related to the praxis dimension of the representation, such as “procedure” (F=6; R=2.333), “team” (F=4; R=4,000), “prescription” (F=4; R=3.750), “management” (F=10; R=2.500), “care” (F=6; R=2.667), “education” (F=5; R=2.600), “decision” (F=6; R=3.000) and “leadership” (F=21; R=3.476). These terms, for the group, associate the object to their practices in internship/residence/technical visits. The peripheral system of the representation has precisely this characteristic, which is to show how the group approaches the object through their practices or how they establish their practices, from the way they resignify the object.

The terms “Cofen/Coren” reflect the imaginary dimension of the representation, based on the professional inspection institutions in Brazil and in the State of Rio de Janeiro, the Federal Nursing Council (Cofen) and the Regional Nursing Council (Coren).

The terms “competence” (F=4; R=4.000), “quality” (F=4; R=3.500), “safety” (F=4; R=3.000), “appreciation” (F=6; R=2.333), “freedom” (F=4; R=1.500), “respect” (F=9; R=3.667), “dedication” (F=4; R=3.000) and “ethics” (F=4; R=3,000), attitudinal dimensions of the representation appearing in the contrast zone and the in the second periphery, seem to reinforce ideas expressed by the presence of the terms “knowledge” and “responsibility” in the probable central core of the representation. The term “support” (F=4; R=2.500), a cognitive dimension present in the contrast zone, refers to the deontological aspects of the nursing profession, which, from the perspective of the group, may be related to the prerequisites for professional autonomy.

In order to confirm certain aspects of the representations produced by the social group, a similarity analysis was also conducted. This is calculated based on the number of co-occurrences between two terms divided by the number of participants, simultaneously. The product of this calculation is the similarity index. For the term “professional nurse autonomy”, 94 of the 171 participants simultaneously evoked two or more words from the four-house board. 

**Figure 3:**
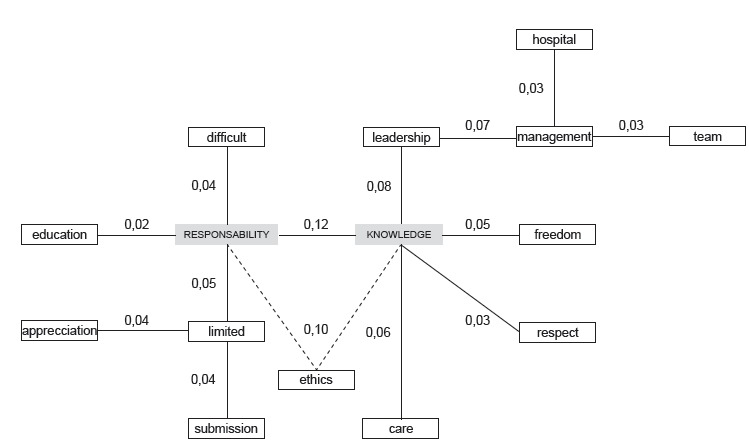
Similarity tree of the cognems produced by the nursing undergraduate student, referring to the object “professional autonomy”. Rio de Janeiro, RJ, Brazil, 2016

Figure 3 shows the similarity tree regarding the object “professional autonomy”, for the entire group of nursing undergraduate students from the federal public universities investigated.

According to the similarity analysis, the cognems that present the highest number of connections in the similarity tree are “responsibility” and “knowledge”, terms already pointed out as possibly central in the four-house board. While not necessarily attesting to the centrality of a given term, the number of connections it establishes with others reinforces the interpretation that such a term is, in fact, a central core in the representation. The term “care” presented only a connection with the term “knowledge”, which puts its centrality in question. Other techniques of collection and analysis, which imply returning to the study scenario, need to be applied to confirm its non-centrality in the structure of the representation.

The terms “responsibility” and “knowledge”, therefore, form two distinct thematic blocks, one mostly attitudinal (responsibility) and the other cognitive (knowledge). This data demonstrates that the social thought of this group regarding professional nurse autonomy is dichotomized between the cognitive/attitudinal dimensions and the actions/knowing/doing dimensions, which complement each other in the constitution of the social thought. The highest index of similarity in the representation, 0.12, was between those two terms, indicating that, because they are inseparable in the social thought of the group, the terms “responsibility” and “knowledge” establish the connection between blocks. 

The cognem “responsibility” is directly or indirectly connected to the terms “limited”, “difficult” and “submission”, three elements of evaluative nature with negative connotation, which indicates the existence of a negative position of the group regarding some of the aspects of the representation. The term “education” is linked to the term “responsibility”, suggesting the idea of ​​a prerequisite for professional autonomy, since it is a great responsibility, in the participants’ opinion. The term “appreciation”, although not directly related to the term “responsibility”, expresses the idea of ​​opposition to the term “limited” , suggesting the existence of internal tension based on the fact that professional autonomy is limited and this can only be reversed through the social appreciation of nurses.

The word “knowledge”, in turn, is strongly linked to terms that are mostly practical in the understanding of the representation, such as “leadership”, “management”, “team”, “care” and “hospital”, the only term of imaginary nature. Here, there is a sense of accomplishment of the professional autonomy in the daily practices of nurses. 

The word “freedom” is strongly connected to the term “knowledge”, leading to the interpretation that the acquisition of technical-scientific knowledge is a propellant to free, independent or autonomous professional practice. The term “ethical” is linked to the terms “responsibility” and “knowledge” by dashed lines so that there would be no closure of these connections in the tree, which is in line with the theoretical perspective of the similarity analysis. In addition, these connections lead to the interpretation that, in the organization of the social thought of the group, the word “ethics” demonstrates a transversality in the two thematic blocks inherent in the reconstruction of autonomy. “Ethics”, therefore, can be expressed in knowledge, as long as there is a continuation of the prerogative of the existence of responsibility.

## Discussion

When comparing the probable central core of the social representation of professional autonomy for students of the first and last period, the permanence of the term “responsibility” is verified. Considering they are students aiming for professionalization, this indicates a challenge for the future care practice, regarding the burden of responsibility of a nurse considered by them as professionally autonomous. This element has representational components of attitudes favorable to the object and can be derived from the growing increase of the nurse’s professional space, although this may be fragile in face of public policy or the profiles of managers in their respective governmental spheres^11^.

It should be noted that the results found in this study are partially different from those obtained from non-nursing health professionals^12^. The central core of the representation of professional nurse autonomy for this population was represented by the elements “care” (similar to the students of the first period) and “responsibility” (similar to the students of the first and last period). However, in the entire structure of the representation of autonomy for non-nursing health professionals, the evocation of “knowledge” was not identified, which may contribute to clarify the reasons for the technical-operational conflicts between nurses and other health professionals. The absence of the term “knowledge” in the structure of the representation by health professionals indicates a dissociation of the nursing profession from the scientific knowledge during the process of symbolization, which may eventually disqualify the profession^12^. For last-period nursing students, who are about to graduate, knowledge not only is present, but has a considerable salience in the central core, with high frequency and low mean order of evocation. Professional nurse autonomy, based on knowledge, is considered a fundamental component of nursing development, an aspect addressed in studies that have emphasized the advantages and importance of autonomous nursing in order to obtain a high quality in the final outcome of patient care, as well as to increase the value of nursing organizations^11^.

Professional nurse autonomy is expressed by undergraduate nursing students, at least in the first instance, as between cognition and attitude, later branching out into other elements that qualify, regulate or clarify it. This is a characteristic of the social thought about the object that hadn’t yet been identified in other studies. These are the two dimensions of the representation that, in addition to organizing the others around themselves, give coherence and consistency to the knowledge regarding professional autonomy contextualized in such an affective and relational profession.

Studies of social representations such as this demonstrate the ability to reveal the codes present in the social thought regarding various themes and, to that end, the structural approach has been highly heuristic^7^ ^-^ ^10^ ^,^ ^12^. Other international authors have focused on the research of technical-scientific instruments to measure and enhance professional nurse autonomy, by formulating dynamic and horizontal health work strategies that allow greater collaboration between nurses and other health professionals (especially doctors)^6^ ^,^ ^13^, provide additions to the nurses’ professional practices in the context of primary care^14^, produce conceptual improvements regarding the clinical role of the nurse^15^ or autonomy-supportive interventions^16^, especially in the hospital environment, or support the relevance of scientific evidences in the care practice of the nurse seeking greater professional autonomy ^(^ ^17^.

However, this research is more aligned with studies addressing the social visibility of nursing and its autonomy. The study is in agreement with several national and international authors ^(^ ^18^ ^-^ ^21^ who argue that, despite the instruments and concepts developed for the professional autonomy of nurses, one of its most important dimension is still the social legitimacy of the nursing practice. The nursing profession is object of several complaints of negligence and malpractice and it is attributed with a certain degree of technical-scientific inferiority, culturally constructed by society. In this scenario, nursing finds an enormous difficulty for establishing itself as an autonomous profession. Under this premise, new psycho-sociological studies are necessary in order to reveal the modes of production and the means of transforming social representations about nursing, nurses and their professional autonomy^12^ ^,^ ^20^ ^-^ ^21^.

There is a strong influence of the nurses’ professional training on their practice in the health work world, since their knowledge, skills and competencies are forged in their undergraduate training. A worldwide trend of transition of nurses from providers of care to prescribers of care is observed and, with this transition, there is a considerable increase in their autonomy and responsibility within health care. This fact is very positive, especially for beginners in nursing practice, such as the participants of this study^22^. The way in which this process occurs can generate positive or negative consequences, both for the professionals and their autonomy and for the quality of the health care provided by them^23^. 

## Conclusion

The social representations of professional nurse autonomy for undergraduate nursing students differ according to the period they are in and their academic experiences in the university. For students of the first period, professional autonomy is resignified in a practical and attitudinal way. Among students of the last period, the knowledge acquired in their know-how stimulate them to assign meaning to professional autonomy conditioning it to the acquisition of scientific knowledge, which contributes to a more cognitive and attitudinal representation. 

In the representational structure of both subgroups, the permanence of responsibility as inherent to the autonomous nursing profession was identified. This is an element that seems to be transverse from the beginning to the end of the training process in the undergraduate nursing course.
